# Optimisation of Hot-Chamber Die-Casting Process of AM60 Alloy Using Taguchi Method

**DOI:** 10.3390/ma17246256

**Published:** 2024-12-21

**Authors:** Tomasz Rzychoń, Andrzej Kiełbus

**Affiliations:** Faculty of Materials Engineering, Silesian University of Technology, ul. Krasińskiego 8, 40-019 Katowice, Poland; andrzej.kielbus@polsl.pl

**Keywords:** AM60, hot-chamber die casting, Taguchi

## Abstract

This paper presents the effect of hot-chamber HPDC (high-pressure die casting) process parameters on the porosity, mechanical properties, and microstructure of AM60 magnesium alloy. To reduce costs, a Taguchi design of the experimental method was used to optimise the HPDC process. Six parameters set at two levels were selected for optimisation, i.e., piston speed in the first phase, piston speed in the second phase, molten metal temperature, piston travel, mould temperature, and die-casting pressure (the pressure under the piston). Signal-to-noise (S/N) ratios were used to quantify the present variations. The significance of the influence of the HPDC parameters was assessed using statistical analysis of variance (ANOVA). The results showed that the die-casting pressure had the most significant influence on the porosity of the AM60 alloy. Moreover, piston speed in the first phase, second phase, and die-casting pressure had the most important effects on tensile strength. It is well known that porosity determines the mechanical properties of die castings; however, in AM60 alloy, changes in the HPDC parameters also contribute to microstructural changes, mainly through the formation of Externally Solidified Crystals.

## 1. Introduction

Magnesium alloys are used in the automotive, aerospace, and electronics industries for their high strength and excellent casting properties. Most components made from magnesium alloy are produced using the die-casting process [1,2]. These are mainly alloys from the Mg–Al system. AZ91 alloy is used for its high strength [3], while AM50/AM60 alloys offer a high ductility [4] and good corrosion and impact resistance at ambient temperature [5]. However, the high-pressure die-casting process these alloys undergo often results in significant porosity in the castings [6].

Light alloy die castings, primarily aluminium and magnesium, are more prevalent in the automotive industry than wrought components [7]. The high-pressure die-casting (HPDC) method is efficient and cost-effective for producing magnesium alloy castings with a high precision. Approximately 90% of cast magnesium alloys are made using HPDC. There are two types of die-casting processes, as follows: cold chamber and hot chamber. In hot-chamber die casting, the injection system is immersed in the liquid metal, while in cold-chamber die casting, the liquid metal is manually or robotically fed into the mould [8].

Casting quality is essential in die-casting production. It is closely related to types of casting defects, making it a crucial factor that requires careful attention [9]. Gas and shrinkage porosities can form in castings due to turbulence and alloy shrinkage, affecting their mechanical properties [10]. Gaseous porosity is a common defect in die castings caused by gas or air entrapment in the mould during the casting process. Shrinkage porosity occurs when the alloy contracts during solidification, decreasing the volume [11]. Shrinkage and gas pores can be identified based on their geometrical features. Shrinkage pores are irregular with many shoulders and have a shape factor of less than 0.4. On the other hand, gas pores have a more circular shape, and their shape ratio is higher than 0.6 [12].

The correct process parameter selection that guarantees a high die-casting quality is a crucial issue for technologists and scientists [13]. The quality of die castings is significantly influenced by various technological factors, including the following [9]:

- the casting speed during the casting cycle,
- the pressure acting on the liquid alloy, the so-called resistance pressure,
- the mould filling time,
- and the liquid metal temperature and mould temperature.

Specific parameters can be controlled, while others are noise factors [14]. A statistical experimental design is a helpful method for effectively planning test numbers to select an appropriate technological parameter set. Experimental design methods are popular in the engineering and scientific community because they can be applied with limited statistical knowledge. The most used design methods include factorial design, response surface methodology (RSM), mixture design, multivariate regression analysis, and the Taguchi method [15,16]. Regarding die-casting parameter optimisation, most research reports are concerned with the Taguchi method [14,15,17,18]. Other casting process optimisation methods appear to be limited, but there are publications on using RSM [19,20,21] or multivariate regression analysis [22]. There is no clear consensus on which of these methods is most effective. In numerous comparisons of the RSM and Taguchi methods, one can find publications indicating that RSM methods are more effective [23,24] or that Taguchi methods are more successful [25,26,27]. In complex optimisation problems, where understanding variable interactions and obtaining accurate answers are crucial, RSM methods are a better solution. On the other hand, the Taguchi method seems more appropriate when the cost of running experiments, minimising the variability of the production process, or the inferior accuracy of the optimisation process are essential to address. For the above reasons, the Taguchi method was chosen in this work.

The Taguchi method is based on searching for nominal values for the controllable factors (process parameters) which satisfy the output characteristic’s maximum compatibility and the lowest noise effect sensitivity. The main benefit of this method is the reduced number of experiments needed to identify the optimal values of the controllable factors. This procedure involves identifying control and noise factors, selecting an orthogonal array (OA) for input variables, and analysing data using the signal-to-noise ratio to measure target achievement. The S/N ratio, also known as the objective function, is inversely proportional to the loss function, so maximising this ratio means minimising losses while improving quality. The three commonly used target functions are “smaller is better”, “higher is better”, and “nominal is best” [28].

Die-casting processes are commonly used to produce castings from aluminium, magnesium, and, less frequently, zinc alloys. In the case of aluminium alloy die castings, most studies using the Taguchi method indicate a critical influence of intensification pressure on the final properties of the casting for both cold-chamber die casting [14,15,17,18] and hot-chamber die casting [17].

When it comes to magnesium alloys, the situation is not as straightforward. Lee et al. [6] analysed the impact of casting parameters on the porosity distribution in the high-pressure die casting of AM50 alloy, which exhibits similarities to AM60 alloy in its microstructure and mechanical properties. They identified gate velocity, intensification pressure, and melt temperature as the most relevant process parameters contributing to a reduced porosity, primarily gaseous porosity.

Park et al. [18] found that the most significant factor in the die-casting process for AM50 alloy is the second piston speed. This speed affects flowability, and increasing it helps to reduce porosity and hardness. In contrast, the pressure and the first piston speed do not significantly affect the microstructure and mechanical properties.

The application of the Taguchi method for optimising the die-casting parameters in AM60 alloy is not widely discussed in the available publications. However, some publications have examined the influence of die-casting parameters, explicitly focusing on cold-chamber die casting. Research has revealed significant information about how casting speed influences the formation and distribution of Externally Solidified Crystals (ESCs), which also plays a crucial role in our study.

Wang et al. [29] showed that, at low casting speeds, large ESCs are present in the structure, while, at high casting speeds, these ESCs tend to become more spherical. Additionally, they found that the slow injection phase (first piston speed) further influences the distribution and level of ESCs’ spheroidization. The distribution and morphology of ESCs also vary based on their location within the casting, such as near the surface, in the core, and near the gating. Wu et al. [30] showed that a lower first piston speed and a longer injection delay time increase ESCs’ size and surface area contribution due to a longer melt holding time in the injection sleeve and a reduction in overheating. In contrast, at a higher second piston speed, ESCs’ exhibit a more granular and circular morphology and are dispersed throughout the casting.

Among the few studies related to the implementation of the Taguchi method in the analysis of magnesium alloy die-casting processes, the research conducted by Sun et al. [31], Wu et al. [13], and Yang et al. [32] is still worth mentioning. Although numerous publications exist on the properties and microstructure of die-cast magnesium alloys, relatively few research studies present the optimisation of the die-casting parameters for these alloys.

This study’s primary objective was to utilise the Taguchi method to determine the optimal technological parameters for the hot-chamber die-casting of AM60 magnesium alloy components. In the literature, the primary casting quality measure, the dependent variable, is casting porosity. This paper investigates whether focusing only on porosity measurements is sufficient, so an additional Taguchi experiment was carried out using the tensile strength *R_m_* as a starting value.

## 2. Experimental Procedure

### 2.1. Material

The study used a magnesium–aluminium casting alloy AM60 with the following composition: Al—6.1%, Mn—0.41%, Zn—0.17%, and Mg—the rest, designed for structural components in the automotive industry. The chemical composition was determined using the SPECTROMAX optical emission spectrometer (AMETEK, Berwyn, IL, USA).

### 2.2. Casting Conditions

**Figure 1:**
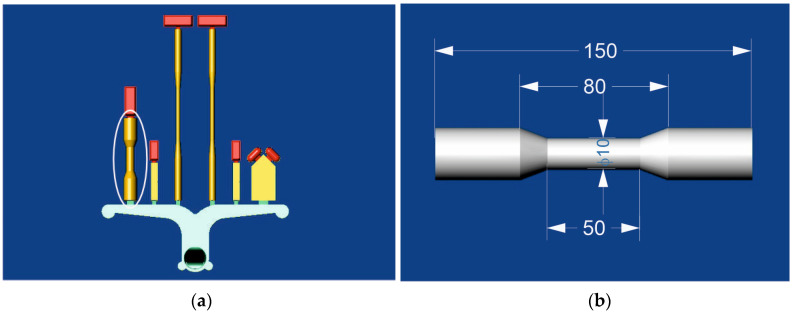
Three-dimensional casting and sample models for porosity and mechanical properties testing, (**a**) casting and (**b**) sample.

The specimens were created using a 280-ton hot-chamber die-casting machine (WEM 280-H, Kędzierzyn-Kożle, Polska). AM60 alloy ingots were preheated in a preheating oven. After reaching approximately 150 °C and ensuring that all moisture was evaporated, they were placed in a melting furnace and heated to about 50 °C higher than the intended casting temperature. From the melting furnace, liquid metal was delivered through transport pipes to the casting furnace, where the entire injection system was immersed. The liquid metal was protected by a mixture of gases, CO_2_ + 1 vol% SF_6_, during melting and holding. Figure 1 shows a 3D cast model and the geometry and dimensions of the strength and porosity test specimen.

In Taguchi’s experiment, eight trials were conducted, each with three repetitions. After each casting parameter change, 6–8 repetitions were performed to stabilise the casting machine.

### 2.3. Radiographic Examination

After the casting process, non-destructive testing was carried out to determine the parameters’ influence on the casting’s porosity. An industrial X-ray scanner, YXLON Y.MU2000-D (Comet Yxlon, Hamburg, Germany), was used for the radiographic tests. A copper anode lamp was used, supplied with a current of 6.25 mA at 160 kV and an HDR filter. All samples were tested according to the ASTM E505 standard (Standard Reference Radiographs for Inspection of Aluminum and Magnesium Die Castings. ASTM: West Conshohocken, PA, USA, 2022).

### 2.4. Porosity Testing Methodology

For each experiment, three samples were prepared. The sample density was calculated according to Archimedes’ principle using the following Equation (1):

(1) $$\rho =\frac{w_{1}}{\left(w_{1}-w_{2}\right)}\cdot \rho_{water}$$

where *ρ*—casting density (g/cm^3^), *w*_1_—casting weight in air (g), *w*_2_—casting weight in water (g), and *ρ_water_*—water density (1 g/cm^3^).

### 2.5. Strength Testing Methodology

A static tensile test was performed according to the ASTM B557 standard (Standard Test Methods for Tension Testing Wrought and Cast Aluminum- and Magnesium-Alloy Products. ASTM: West Conshohocken, PA, USA, 2015) using an Instron 4469 testing machine (Instron, Norwood, MA, USA). The machine had a force range of 30 kN and operated at a strain rate of 10 mm/min. Elongation measurements were taken with an extensometer featuring a measuring base of 60 mm.

### 2.6. Microscopic Testing Methodology

The microstructure of the AM60 alloy was analysed by light microscopy (LM) using an Olympus GX-71 microscope (Olympus, Tokyo, Japan) and scanning electron microscopy (SEM) using an FE SEM Hitachi S-3400N (Hitachi, Tokyo, Japan) scanning electron microscope. Microstructure observations were performed on unetched samples to reveal their porosity. To reveal the microstructure of the AM60 alloy, samples were etched in a reagent containing 3 mL of HNO_3_ and 97 mL of C_2_H_5_OH. Quantitative metallography was used to determine the α-Mg grain size.

### 2.7. Taguchi Methodology

**Figure 2:**
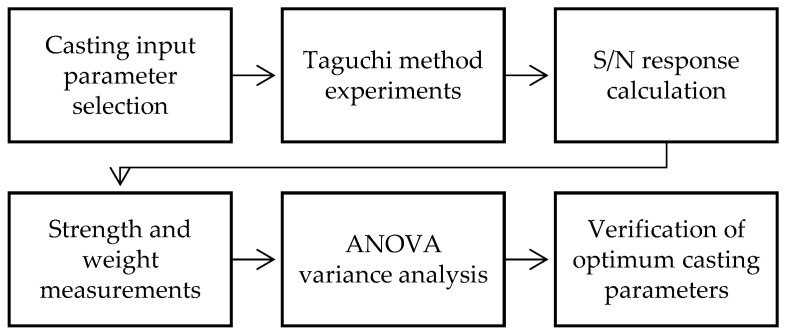
Experimental methodology.

This research aims to analyse the influence of hot-chamber die-casting variables on the optimal combination of control factors (parameters) to maximise the tensile strength and minimise the porosity in AM60 alloy. The methodology used in the study is shown in Figure 2.

The output response variability was determined by signal-to-noise ratio (*S*/*N*) analysis. The signal-to-noise (*S*/*N*) ratio was derived from the quadratic loss function. Depending on the type of property being tested, different *S*/*N* ratios may apply, including “lower is better”, “higher is better”, and “nominal is best”. Tensile strength is a “bigger is better” quality characteristic, aiming to maximise strength properties. For the criterion “higher is better”, the objective is to maximise the occurrence of the required product characteristics, which is enabled by the following criterion function (2):

(2) $$\eta =-10\log\left[\frac{1}{n}\sum_{i=1}^{n}\frac{1}{y_{i}^{2}}\right]$$

where:

*i*—number of simulation design parameters;

*η*—signal-to-noise (*S*/*N*) ratio;

*n*—number of simulation repetitions under the same design parameter conditions,

*y*—results of measuring

Porosity is a “smaller is better” quality characteristic, aiming to minimise porosity and increase mechanical properties. In the “smaller is better” criterion, the aim is to minimise the occurrence of unwanted product characteristics, which is enabled by the following criterion function (3):

(3) $$\eta =-10log\left[\frac{1}{n}\sum_{i=1}^{n}y_{i}^{2}\right]$$

where:

*i*—number of simulation design parameters;

*η*—signal-to-noise (*S*/*N*) ratio;

*n*—number of simulation repetitions under the same design parameter conditions,

*y*—results of measuring

In addition, a statistical analysis of variance (ANOVA) was performed to evaluate the significance of the process variables on the output characteristics. By utilising *S*/*N* and ANOVA analyses, the optimal experimental conditions were determined. Finally, a verification experiment was conducted to confirm the validity of the optimal process parameters identified during the parameter design and to assess whether porosity was the only significant factor influencing the strength of AM60.

## 3. Results and Discussion

**Figure 3:**
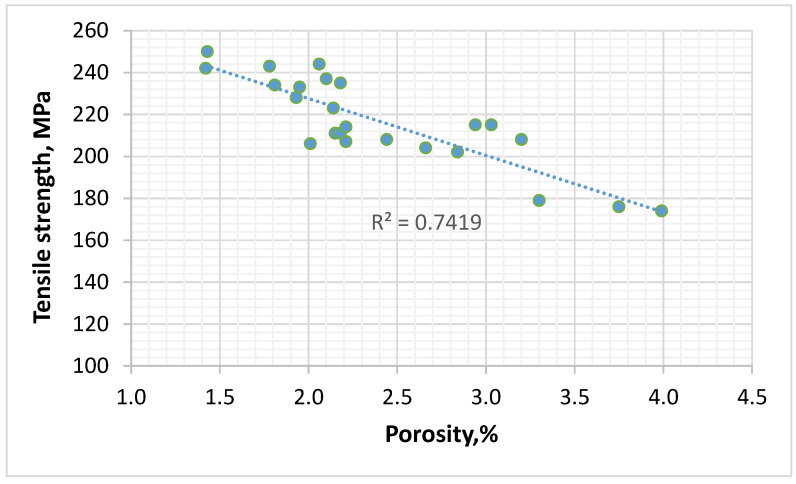
The influence of porosity on the mechanical properties of AM60 alloy.

It is commonly known that increasing the porosity of materials causes the deterioration of mechanical properties due to a reduction in the active cross-section transferring the loads. This is similar in the case of the AM60 alloy after die casting (Figure 3). It is worth emphasising that the correlation between porosity and tensile strength is not too high, and the determination coefficient is only *R*^2^ = 0.74. On the one hand, this is due to measurement inaccuracies and the uneven distribution of pores in the sample volume. On the other hand, with minor changes in porosity (from 1.5 to 4%), which were observed during the hot-chamber die casting of the AM60 alloy with different parameters, the mechanical properties will also be influenced by microstructural factors, i.e., the homogeneity of the microstructure, the volume fraction of the Mg_17_Al_12_ phase, the grain size of the α-Mg solid solution, or solution strengthening.

### 3.1. Taguchi Method

**Figure 4:**
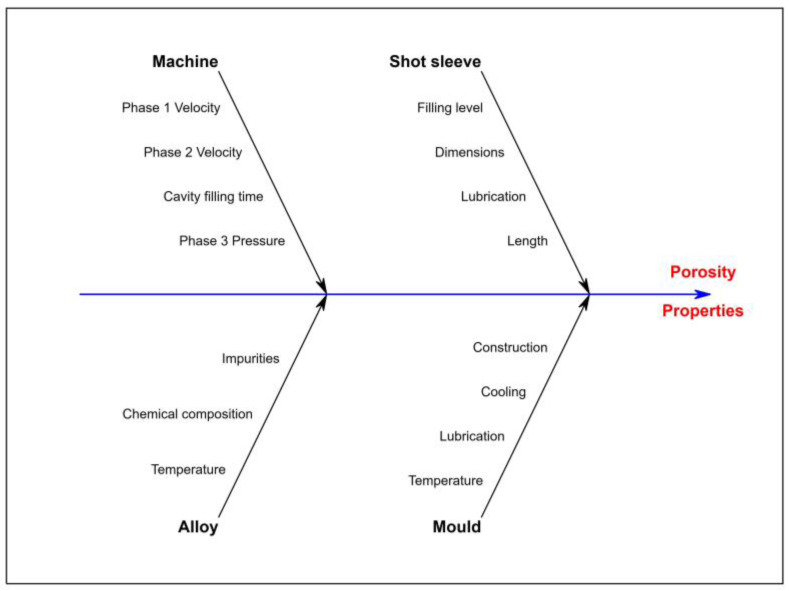
Ishikawa diagram for hot-chamber die casting.

The Taguchi method analysis began with selecting the factors influencing the quality of the castings produced using the hot-chamber die-casting method. The technological parameters of the process were divided into the following four categories: (a) parameters related to the casting machine, (b) parameters related to the injection system, (c) parameters of the liquid metal, and (d) parameters related to the casting mould. The Ishikawa diagram schematically presents the most important parameters determining the quality of castings (Figure 4).

The selection of parameters and their levels was made based on preliminary studies, data from the literature, and the technological capabilities of the high-pressure machine. These are as follows:

A—metal temperature—the temperature of the liquid metal injected into the mould,
B—mould temperature—the mould cavity temperature,
C—piston speed in the first phase—the slow injection phase,
D—piston speed in the second phase—the phase of filling the actual mould cavity,
E—piston travel in the second phase—the path covered by the piston in the second phase of metal injection,
F—pressure—the pressure acting on the alloy in the third injection phase.

Two levels were adopted for each selected parameter (Table 1), while the remaining ones were kept constant throughout the experiment. The total number of degrees of freedom for the six factors (parameters) at two levels was five. Therefore, an orthogonal table L8 (Table 2) was selected, specifying eight experiments and seven columns. Possible interactions between independent variables were omitted in the analysis.

### 3.2. Porosity Assessment Using the Taguchi Method

The signal-to-noise (*S*/*N*) values were calculated using “smaller-is-better” performance characteristics, and the calculation results are presented in Table 2. As can be seen, the lowest porosity (the highest *S*/*N* values) was observed for trial no. 7. The response table (Table 3) shows that pressure had the most significant effect on porosity, and to obtain the lowest porosity, the process should be carried out at its maximum values. Increasing the piston speed during the first phase and second phase and the piston path in the second phase contributed to reducing the porosity of the castings. The mould and liquid metal temperature exerted the slightest effect on the porosity of the castings due to the low values of the *R* coefficient.

The Taguchi analysis provides information on which casting parameters used had the most significant influence on porosity. Still, it did not answer the question of the percentage contributions of individual parameters to porosity, nor did it provide an answer to the question of which factors were statistically significant. This information can be obtained from ANOVA analysis (Table 4), performed for the *S*/*N* values obtained from the orthogonal array (Table 2).

Fischer *F* significance tests for the above parameters were conducted for the 95% confidence level. As can be concluded from Table 4, only the die-casting pressure had a significant effect on porosity (*p* = 0.045). The contribution of pressure to porosity was the largest (42.9%) among the parameters used. For the piston speed and travel in the second phase, a significant contribution ratio of over 20% was obtained. However, from a statistical point of view, the hypothesis of a significant influence of these factors should be rejected. On the other hand, the *p* probability values were close to the assumed significance level. Therefore, it seems reasonable to control these parameters and include them in further stages of process optimisation. The piston speed in the first phase, despite a contribution of over 10%, was also statistically insignificant according to the results of the *F* test. The influences of the liquid metal temperature and the mould temperature in the assumed range of values were marginal. It is worth emphasising that ANOVA calculations were also performed after pooling the non-significant parameters, often recommended in Taguchi analysis. In this case, the *F*-test results indicated a complete lack of significance, meaning that the tested casting parameters did not affect the porosity.

### 3.3. Evaluation of Tensile Strength Using the Taguchi Method

The influence of parameters on the tensile strength *R_m_* was also analysed using the L8 orthogonal array (Table 5) for the same samples for which the porosity was measured using the Archimedes method. In this case, calculations were used by the “higher is better” criterion.

It can be concluded from Table 6 that the most significant influence on tensile strength was the piston speed in the second phase, followed by the pressure and piston speed in the first phase. The ANOVA analysis of variance (Table 7) indicates that these parameters significantly (*p* < 0.05) affected the strength properties of the die castings made of the AM60 alloy. According to the ANOVA analysis, the remaining casting parameters had a minor contribution to the tensile strength (<6%) and were statistically insignificant.

### 3.4. Verification Experiment

The Taguchi method indicated a varied effect of the hot-chamber die-casting parameters on porosity and tensile strength (Table 4 and Table 7). The die casting pressure most significantly affected the porosity, while the tensile strength *R_m_* was most significantly affected by the piston speed in the second phase and the pressure and piston speed in the first phase. In the last stage of optimising the die-casting parameters, verification was carried out by hot-chamber die casting with optimal parameters, which should guarantee the highest tensile strength. According to the *S*/*N* ratio response table (Table 6), setting all parameters at level 2 should ensure the highest strength within the assumed parameter range. Controlling the liquid metal temperature and the mould temperature can be omitted entirely due to the marginal influences of these factors. From a practical point of view, setting these parameters at level 1 is more beneficial, as it will reduce the costs related to maintaining a lower temperature of the liquid metal (A) and the mould (B). Despite differences in the assessment of the significance of the influences of individual parameters on strength and porosity, setting all casting parameters at level 2, except for the mould temperature, should also provide the lowest porosity (Table 3). Similarly to the analysis of the parameters influencing strength, the contributions of the liquid metal temperature and mould temperature to porosity were also found to be negligible. Finally, the verification experiment was carried out with the parameter levels presented in Table 8.

The results of the verification experiment can be considered as satisfactory (typical mechanical properties for die-cast AM60 alloy are in the range of 230–260 MPa [33]), and the highest tensile strength (*R_m_* = 250 MPa) and the lowest porosity (1.62%) were obtained for new parameter settings.

The values obtained in the verification experiment were compared with the predicted values for the optimal parameters. To estimate the tensile strength and porosity under optimal die-casting conditions, the following equation can be used [34]:

η_opt_ = η + (A1 − η) + (B1 − η) + (C2 − η) + (D2 − η) + (E2 − η) + (F2 − η) (4)

where:

η_opt_ is the predicted average *S*/*N* ratio for the *R_m_* and porosity,

η is the overall experimental mean of the *S*/*N* ratio,

and A1, B1, C2, D2, E2, and F2 are the *S*/*N* ratio values for the recommended level of the process parameters (Table 3 and Table 6).

The predicted values calculated from Equation (4) were compared with the experimental values in Table 9, and the error percentage between them was calculated.

The results fell short of the expected values calculated from the Taguchi experiment. It is common practice for an optimisation process that, if the average error deviation is less than 10%, then the optimisation can be considered as valid for the model to be accepted. The obtained optimal tensile strength value was nearly like the verification experimental trial value in Table 8, confirming that the optimal conditions were satisfactory. For porosity, the percentage error (9.5%) was much higher; therefore, using this feature in industrial practice as the only indicator for assessing the quality of castings may lead to an erroneous estimation of the optimal die-casting parameters. The significant deviations between the expected values obtained from the Taguchi model and the experimental values were due to the large dispersion of porosity measurement results in subsequent replications (three replications were used for each experimental trial). For tensile strength, the dispersion of results measured with the variability index was 1–3%, and for porosity, this was in the range of 4–18%. Such results indicate that the porosity was not sufficiently sensitive to changes in the process parameters.

### 3.5. Microstructure of Die-Cast AM60 Alloy

**Figure 5:**
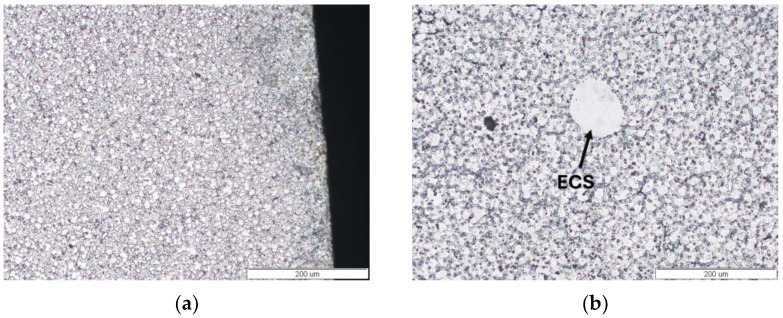
Microstructure of die-cast AM60 alloy obtained under optimal hot-chamber die-casting parameters reported in Table 8; at the surface (**a**) and in central zone (**b**).

**Figure 6:**
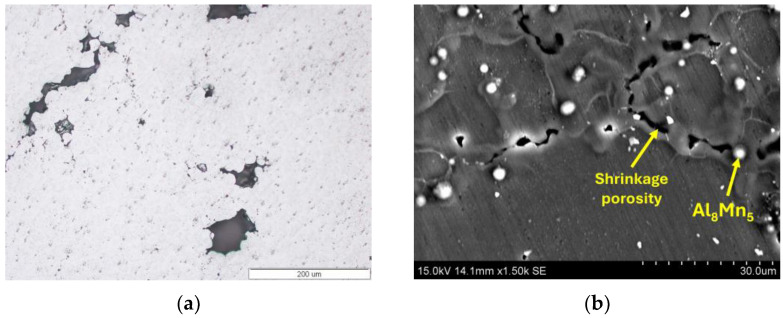
Gas (**a**) and shrinkage (**b**) porosity in die-cast AM60 alloy.

**Figure 7:**
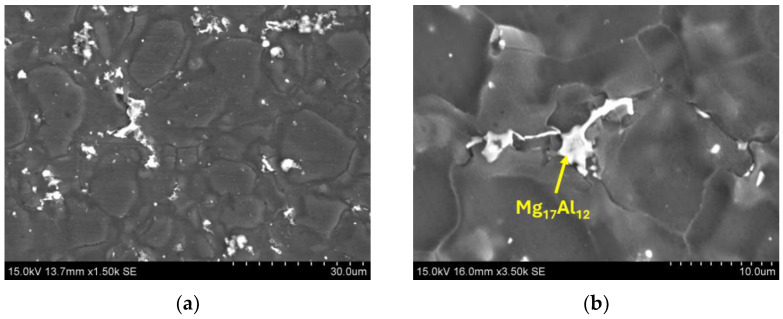
Intermetallic compounds in die-cast AM60 alloy (**a**), morphology of the Mg_17_Al_12_ phase (**b**).

After experiments using the Taguchi method, the microstructure of the AM60 alloy was characterised using light microscopy (LM) and scanning electron microscopy (SEM). The microstructure after hot-chamber die casting with the optimal parameters (Table 8) was typical for high-pressure die castings. It was characterised by refined grains of the α-Mg solid solution and the presence of “skin” with highly refined grains near the surface (Figure 5a). The grain size changes in the cross-section and at the surface were 9.7 ± 0.4 μm and in the central part were 19.6 ± 0.8 μm (Figure 5b). In the central zone of the casting, large equiaxed grains of α-Mg solution with a diameter of up to approx. 80 μm were also visible, classified as Externally Solidified Crystals (ESCs). Another characteristic feature of high-pressure die castings is gas and shrinkage porosity. Gas pores usually have a regular shape and are in the central part of the casting. In contrast, shrinkage pores are mainly located in the interdendritic areas (Figure 6). Aluminium dissolves in magnesium to form an α-Mg solid solution, contributing to solution strengthening and creating an intermetallic Mg_17_Al_12_ phase in the interdendritic areas and an Al_8_Mn_5_ phase located both inside the grain of the solid solution of α-Mg and at the grain boundaries (Figure 6 and Figure 7).

As mentioned, the Taguchi method indicated a varied effect of the hot-chamber die-casting parameters on porosity and tensile strength (Table 4 and Table 7). This may cause a problem for technologists designing die-casting processes to select an appropriate casting quality factor. Therefore, the following questions arise: (i) whether the differences result from the limitations of the Taguchi method; (ii) whether the differences are caused by an incorrectly adopted range of parameters for optimisation; and (iii) are there any microstructural factors (e.g., grain size, Al content in Mg) that affect the strength of the AM60 alloy.

This part of the article attempts to answer the following question: how do the technological process parameters affect the microstructure of the AM60 alloy, and does this affect the tensile strength? For this purpose, the castings obtained in trial no. 3 and trial no. 7 were selected for further studies (Table 2). A similar porosity was obtained (1.76% and 1.72%, respectively), while samples from trial no. 3 showed a higher tensile strength (Table 3, trial no. 3—246 MPa and trial no. 7—235 MPa). Suppose that we consider the insignificance of the influence of the liquid metal temperature and the mould temperature on the resulting features. In that case, both experiments differ regarding the piston speed in the first phase and the piston travel in the second phase.

**Figure 8:**
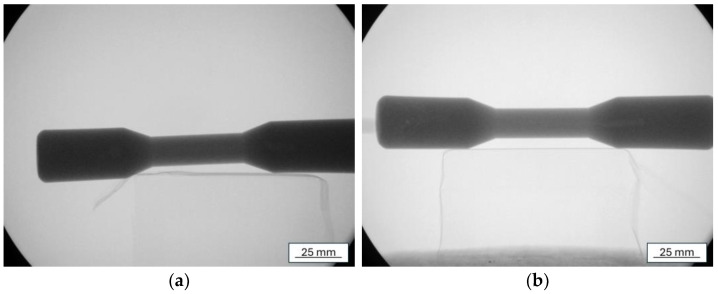
Radiographic examination of castings: trial no. 3 (**a**) and trial no. 7 (**b**).

**Figure 9:**
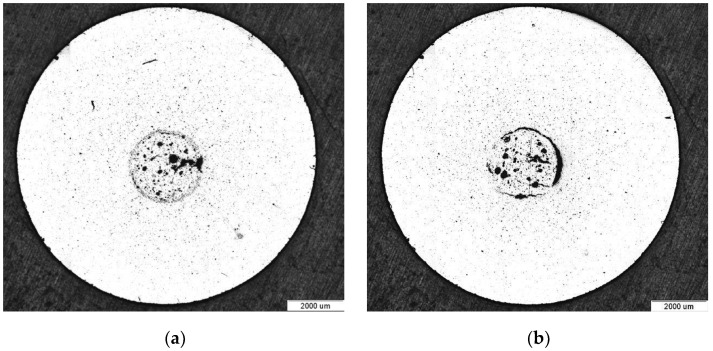
Porosity in samples cast with the parameters: (**a**) trial no. 3 and (**b**) trial no. 7.

The porosity measurements of both samples using the Archimedes method (Table 2) and radiographic examinations indicated a similar porosity (Figure 8). The pore distribution on the cross-section was also comparable in both castings. The highest porosity was in the central part of the sample (Figure 9). In the case of samples cast in trial no. 7, the average surface area of the flat pore cross-section was slightly larger (Ā = 1301 μm) compared to the samples from trial no. 3 (Ā = 1126 μm).

**Figure 10:**
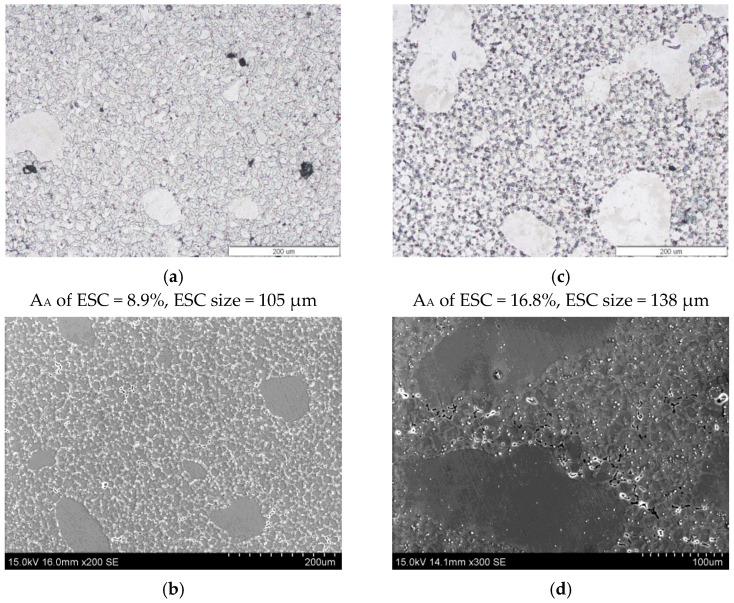
Externally Solidified Crystals in the die-cast AM60 alloy cast using the parameters of experiment no. 3 (**a**,**b**) and cast using the parameters of experiment no. 7 (**c**,**d**). Area fraction of ESCs, ESC size—mean size of ESCs.

Observations of the microstructure of the AM60 alloy provided important information. Samples with a lower tensile strength had a larger volume fraction of Externally Solidified Crystals—ESCs (Figure 10c,d) than those with a higher tensile strength (Figure 10a,b). This was also confirmed by the quantitative analysis of the grain size, which showed that the area fraction A_A_ of large grains (ESC), as well as their grain size, was larger in castings with a lower strength (trail no. 7) (Figure 10).

Al in the AM60 alloy participated in the formation of Mg_17_Al_12_ and Al_8_Mn_5_ intermetallic phases and dissolved in the α-Mg solid solution. The volume fraction of the Mg_17_Al_12_ phase may affect the mechanical properties of the alloy. However, in this case, the differences in the content of the Mg_17_Al_12_ phase between trial no. 3 and trial no. 7 were not significant. No significant differences were found in the content of Al dissolved in the α-Mg solid solution.

After analysing the results, Externally Solidified Crystals (ESCs) significantly influenced the mechanical properties. Increasing their content and diameter would contribute to a decrease in strength, which may be mainly related to the reduction in the piston speed in the first phase. ESCs are equiaxed dendrites that nucleate and grow in the liquid metal, filling the shot sleeve before its injection into the die cavity (first phase of the piston) [35]. When the molten metal is held in the sleeve, especially in the first injection phase (slow injection phase), the overheating of the liquid alloy can be reduced, facilitating the nucleation of ESCs [30]. The volume fraction of ESCs formed in the shot sleeve can be reduced by increasing overheating before injection, for example, by increasing the initial melt and injection nozzle temperatures [35]. The piston speed of the first and second phases is also important; piston acceleration will help to reduce the residence time of the liquid metal in the shot sleeve. A lower piston speed increases ECSs’ size and volume fraction due to the longer time it takes to hold the melted alloy in the shot sleeve, promoting nucleation and ESC growth. It follows that, with the increase in the speed of the first injection phase, the volume fraction of ESCs and pores is much smaller [36]. On the other hand, with the increase in the piston speed in the second injection phase, the melting and fragmentation of ESCs are accelerated [30].

In the case of the hot-chamber die casting of AM60 alloy, the development of ESCs consists of the following four stages: nucleation, growth, remelting, and fragmentation. In the first injection phase, when the liquid metal is held in the shot sleeve, the melt overheating is reduced, and ESC nucleation and growth occur. At low piston speeds in the first phase, there will be a tendency to form large ESCs in the alloy’s microstructure. This is also confirmed by the results of the Taguchi analysis, which indicate that the piston speed in the first phase significantly affects the alloy strength (Table 7). In the second phase, the high speed of the liquid metal causes molten metal turbulence, which, in consequence, leads to the remelting and fragmentation of ESCs and obtaining finer and more uniformly distributed ESC grains. For these reasons, the piston speeds in the first and second phase are important for obtaining a high tensile strength. The die-casting pressure is also a critical factor in determining the mechanical properties of the AM60 alloy due to improving the feeding ability of the liquid alloy, which contributes to reducing porosity and grain refinement.

## 4. Conclusions

This paper used the Taguchi method to determine the most beneficial parameters of hot-chamber pressure casting on the strength of AM60 alloy castings. The following conclusions can be drawn:

1. The die-casting pressure exerts the most significant influence on the porosity of die-cast AM60 alloy. The contribution of pressure to porosity is 42.9%, with a significance level of *p* = 0.045. The remaining factors used in this experiment are not statistically significant.
2. The die-casting pressure and the piston speed in the first and second phases statistically significantly influence the tensile strength. The highest contribution to tensile strength is the piston speed in the second phase (46.7%, *p* = 0.032). The contributions of the piston speed in the first phase and the pressure to tensile strength are 21.6% (*p* = 0.047) and 24.6% (*p* = 0.044), respectively.
3. Besides die-casting pressure, the piston speed in the first and second phases is of great importance for obtaining castings with high mechanical properties, which contributes to a reduction in the fraction and refinement of Externally Solidified Crystals.
4. The Taguchi method for optimising hot-chamber die-casting parameters should not be based only on assessing porosity. Other quality measures, e.g., mechanical properties or the assessment of ESCs’ size and content, should be included in the design of optimal die-casting parameters.
5. The validation experiment results for the optimal die casting parameters (*R_m_* = 150 MPa) show good agreement for the tensile strength (predicted value *R_m_* = 154 MPa), giving an error value of 2.7%. For porosity, a percentage error of 9.5% is obtained. The predicted porosity for the optimal parameters is 1.48%, and the obtained value in the verification test is 1.62%.

## Figures and Tables

**Table 1:** Controlled parameters and their levels.

Designation		Parameter	Level 1	Level 2
Tm (°C)	A	Molten temperature	620	650
Td (°C)	B	Mould temperature	160	200
V1 (mm/s)	C	Piston speed in the 1st phase	50	80
V2 (mm/s)	D	Piston speed in the 2nd phase	300	450
L2 (mm)	E	Piston travel in 2nd phase	75	120
P (MPa)	F	Die-casting pressure	20	80

**Table 2:** L8 orthogonal array with porosity measurement results and calculated *S*/*N* values.

Trial	A	B	C	D	E	F	No. 1	No. 2	No. 3	Porosity, % (Mean)	*S*/*N*
1	2	2	1	2	1	1	2.84	3.03	3.20	3.02	−9.6200
2	2	2	1	1	2	2	2.01	2.15	2.18	2.11	−6.5047
3	2	1	2	2	1	2	1.78	2.06	1.43	1.76	−4.9863
4	2	1	2	1	2	1	2.44	2.66	2.21	2.44	−7.7555
5	1	2	2	2	2	1	2.10	1.95	2.18	2.08	−6.3565
6	1	2	2	1	1	2	2.14	2.94	2.21	2.43	−7.8073
7	1	1	1	2	2	2	1.93	1.81	1.42	1.72	−4.7796
8	1	1	1	1	1	1	3.99	3.30	3.75	3.68	−11.3431

**Table 3:** Response table of *S*/*N* ratios showing effects of hot-chamber die-casting parameters on porosity of AM60 alloy.

Level	A	B	C	D	E	F
1	−7.57	−7.22	−8.06	−8.35	−8.44	−8.77
2	−7.22	−7.57	−6.73	−6.44	−6.35	−6.02
*R*	0.355	0.356	1.335	1.917	2.090	2.749
Rank	6	5	4	3	2	1

**Table 4:** Results of ANOVA analysis for the porosity of the die-cast AM60 alloy (significance level α = 0.05).

Factors	SS	DOF	Variance	*F*	*p*	Contribution, %
A	0.252062	1	0.25	3.38	0.316966	0.7
B	0.253459	1	0.25	3.40	0.316228	0.7
C	3.566824	1	3.57	47.89	0.091358	10.1
D	7.350395	1	7.35	98.70	0.063866	20.8
E	8.737010	1	8.74	117.32	0.05861	24.8
F	15.117319	1	15.12	202.99	0.04461	42.9
Error	0.074475	1	0.07			
Total	35.35	7	5.05			

**Table 5:** L8 orthogonal array with tensile strength measurement results and calculated *S*/*N* values.

Trial	A	B	C	D	E	F	No. 1	No. 2	No. 3	*R_m_*, MPa (Mean)	*S*/*N*
1	2	2	1	2	1	1	202	215	208	208	46.37
2	2	2	1	1	2	2	206	211	211	209	46.42
3	2	1	2	2	1	2	243	244	250	246	47.80
4	2	1	2	1	2	1	208	204	207	206	46.29
5	1	2	2	2	2	1	237	233	235	235	47.42
6	1	2	2	1	1	2	223	215	214	217	46.74
7	1	1	1	2	2	2	228	234	242	235	47.40
8	1	1	1	1	1	1	174	179	176	176	44.92

**Table 6:** Response table of *S*/*N* ratios showing effects of hot-chamber die-casting parameters on tensile strength of AM60 alloy.

Level	A	B	C	D	E	F
1	46.62	46.61	46.28	46.09	46.46	46.25
2	46.72	46.74	47.06	47.25	46.88	47.09
*R*	0.098	0.130	0.787	1.156	0.423	0.839
Rank	6	5	3	1	4	2

**Table 7:** Results of ANOVA for the tensile strength of the die-cast AM60 alloy (significance level α = 0.05).

Factors	SS	DOF	Variance	F	*p*	Contribution, %
A	0.019222	1	0.02	2.88	0.339	0.3
B	0.033681	1	0.03	5.05	0.267	0.6
C	1.237323	1	1.24	185.50	0.047	21.6
D	2.673800	1	2.67	400.87	0.032	46.7
E	0.358260	1	0.36	53.71	0.086	6.3
F	1.408275	1	1.41	211.13	0.044	24.6
Error	0.006670	1	0.01			
Total	5.74	7	0.82			

**Table 8:** Porosity and tensile strength measurement results obtained after the verification experiment.

Trial	A	B	C	D	E	F	1	2	3	*R_m_*, MPa (Mean)	1	2	3	Porosity %
1	1	1	2	2	2	2	248	253	250	250	1.74	1.62	1.49	1.62

**Table 9:** Predicted and experimental values of tensile strength and porosity under optimal die-casting parameters of AM60 alloy.

Parameter	Predicted	Experimental	Error, %
*R_m_*, MPa	254	250	2.7
Porosity, %	1.48	1.62	9.5

## Data Availability

The original contributions presented in this study are included in the article. Further inquiries can be directed to the corresponding author.
